# Specialist palliative care services for adults with advanced, incurable illness in hospital, hospice, or community settings—protocol for a systematic review

**DOI:** 10.1186/s13643-015-0121-4

**Published:** 2015-09-25

**Authors:** Jan Gaertner, Waldemar Siemens, Gerd Antes, Joerg J Meerpohl, Carola Xander, Guido Schwarzer, Stephanie Stock, Gerhild Becker

**Affiliations:** Department of Palliative Care, University Medical Center, Robert-Koch-Straße 3, 79106 Freiburg, Germany; German Cochrane Centre, Medical Center, University of Freiburg, Freiburg, Germany; Institute of Medical Biometry and Informatics, Medical Center, University of Freiburg, Freiburg, Germany; Institute for Health Economics and Clinical Epidemiology, Cologne University Hospital, Cologne, Germany

**Keywords:** Palliative care, Advanced illness, Quality of life, Effect, Systematic review, Randomized controlled trial

## Abstract

**Background:**

Specialist palliative care (SPC) interventions aim to relieve and prevent suffering in the physical, psychological, social, and spiritual domain. Therefore, SPC is carried out by a multi-professional team with different occupations (e.g., physician, nurse, psychologist, and social worker). Remaining skepticism concerning the need for SPC may be based on the scarcity of high-quality evaluations about the external evidence for SPC. Therefore, we will conduct a systematic review according to Cochrane standards to examine the effects of SPC for adults with advanced illness.

**Methods/design:**

The comprehensive systematic literature search will include randomized controlled trials (RCTs) and cluster RCTs. We will search the databases MEDLINE, EMBASE, Cochrane Central Register of Controlled Trials (CENTRAL), and PsycINFO.

Patients must be adults suffering from life-limiting diseases. Proxy and caregiver outcomes will not be assessed in order to ensure a clear and well-defined research question for this review. Interventions may be in an in- or outpatient setting, e.g., consulting service, palliative care ward, and palliative outpatient clinic. In line with the multi-dimensional scope of palliative care, the primary outcome is quality of life (QoL). Key secondary outcomes are patients’ symptom burden, place of death and survival, and health economic aspects. Subgroup analysis will assess results according to cancer type, age, early vs not early SPC, site of care, and setting.

Analysis will be performed with the current RevMan software. We will use the Cochrane Collaboration risk of bias assessment tool. The quality of evidence will be judged according to the Grading of Recommendations Assessment, Development, and Evaluation (GRADE) approach.

**Discussion:**

The available evidence will be summarized and discussed to provide a basis for decision-making among health care professionals and policy makers. For SPC, we believe that multi-professional care is of utmost importance. Therefore, single-profession interventions such as physician consultations will not be included. Based on the multi-dimensional scope of palliative care, we chose QoL as the primary outcome, despite an expected heterogeneity among the QoL outcomes. We consider unidimensional endpoints such as “pain” for the physical domain to be inadequate for capturing the true scope of (S)PC (i.e., QoL) as defined by the World Health Organization.

**Systematic review registration:**

PROSPERO CRD42015020674

**Electronic supplementary material:**

The online version of this article (doi:10.1186/s13643-015-0121-4) contains supplementary material, which is available to authorized users.

## Background

Palliative care aims to relieve and prevent suffering in the physical, psychological, social, and spiritual domain [1] for people with advanced and life-limiting diseases. Palliative care has often been misconstrued as terminal or end-of-life care or as the provision of disease-modifying interventions for incurably ill patients [2, 3]. Therefore, it is important to prevent such misunderstandings in the interpretation of research publications by referring to an existing definition for palliative care when outlining the scope of research [2, 3]. For this review, the definition of the World Health Organization (WHO) provides a common basis [1].

It is important to point out that the WHO defines palliative care as an *approach* that is delivered by all physicians (regardless of their discipline) caring for a patient (i.e., general, primary, or basic palliative care) [1, 3]. Beyond this, the WHO also supports the development and widespread availability of specialist palliative care (SPC) or speciality palliative care. Normally, SPC is provided by multi-professional teams on inpatient wards or as consultation teams within hospitals in outpatient clinics and as palliative home care. [4]. Recently, SPC has become increasingly available. For example, palliative care consultation services/teams (i.e., mostly consisting of a physician, an experienced nurse, a psychologist, and a social worker) are being rapidly implemented, especially in large academic centers [5]. In the USA, the proportion of academic medical centers providing a palliative care consultation service has increased fivefold (from 15 to 75 %) during the last decade [5]. In contrast, the provision of inpatient palliative care wards is not very well-established in the USA, though there is a comparatively long tradition in Western Europe [6].

### Description of the condition

Palliative care aims at improving “quality of life of patients and their families facing the problems associated with life-threatening illness, through the prevention and relief of suffering by means of early identification and impeccable assessment and treatment of pain and other problems, physical, psychosocial, and spiritual” [7]. Among patients with (advanced) cancer, common physical (e.g., nausea) and psychosocial (e.g., depression, anxiety) needs as well as economic concerns are not adequately addressed [8]. In addition, hospital staff often reports not having enough time to care for dying patients and the majority does not feel well-prepared for this task according to a representative survey [9]. Moreover, intensive or aggressive therapies at the end of life (e.g., chemotherapy) have a considerable financial impact on patients/patient’s family and society [10]. Aggressive therapies at the end of life might not even be beneficial for patients’ quality of life (QoL) [10, 11].

### Why it is important to do this review

Even when SPC services are available, some physicians hesitate to initiate a specialist palliative care visit for their patients [12–15]. The scarcity of high-quality evaluations (e.g., systematic reviews) about the effectiveness of SPC could be one reason for this [15]. In fact, there is an unclear evidence base for most of the recommendations for the integration of SPC [16]. To bridge this gap, it is crucial to assess the evidence base of SPC and identify the most effective components of SPC [17–19].

To more adequately address the unmet needs of palliative care patients, the availability of SPC in addition to basic (or general) palliative care [1, 3] is being increasingly advocated [3, 4, 13, 20]. SPC has also emerged as a medical specialty and is available in addition to general palliative care in many different settings [20–22]. Currently, the integration of SPC into the care of patients with various advanced and incurable illnesses (e.g. cancer, heart failure, neurodegenerative diseases) is recommended [3, 16, 23–26]. The integration of SPC is said to increase patients’ QoL, reduce patient and family distress and the aggressiveness of care and health care costs, and may even prolong life [3, 27, 28].

We used the Preferred Reporting Items for Systematic review and Meta-Analysis Protocols (PRISMA-P) 2015 checklist in order to ensure completeness of the protocol (Additional file 1). The primary objective of this systematic review is to examine the effects of SPC services for adults with advanced illness on QoL.

## Methods/design

### Criteria for considering studies for this review

#### Types of studies

According to the Cochrane Handbook [29] and based on the availability of RCTs found by comparable reviews [3, 30], we chose to include the following types of studies:

1. Double-, single-blind, or not blinded RCTs
2. Cluster RCTs

We decided to exclude all other study designs because none of the three main reasons for including non-randomized studies stated in the Cochrane Handbook (chapter 13.1.2) were applicable [29].

We will include full journal publications, results from clinical trial platforms (if not stated in journals), and abstracts with sufficient data for analysis of otherwise unpublished clinical trials.

We define “randomized” as studies that are described by the authors as “randomized” anywhere in the manuscript, except in cases where the term is misused (e.g., referring to a random sample).

#### Types of participants

Adult in- or outpatients (aged 18 years or older) with advanced illness, regardless of whether malignant or non-malignant (e.g., metastatic lung cancer, glioblastoma, heart or renal failure, neurodegenerative diseases, etc.), who are receiving SPC (as described below) were included. “Advanced illness is defined as when one or more conditions become serious enough that general health and functioning decline and treatment begins to lose effect – a process that extends to the end of life” [31].

### Description of the intervention

#### SPC (synonyms: specialized or specialty palliative care)

Normally, SPC is provided by multi-professional teams on inpatient wards or by consultation teams within hospitals, in outpatient clinics, and as palliative home care [4].

#### How the intervention might work

SPC services are provided by health care professionals who usually have received a higher degree of *palliative care training*. Another advantage of SPC services is that they can *focus on alleviating and preventing suffering* without being primarily responsible for the disease-modifying therapies (e.g., chemotherapy, cardiovascular interventions) [16, 23–26]. Also, if such *conjoint and interdisciplinary cooperation* of the primarily responsible disciplines (e.g., cardiology, oncology, or neurology) with SPC services is well coordinated, the joining of the expertise, vigilance, and workforce of both disciplines may improve efficacy and safety of care. These aspects along with the *multi-professional care model* allow SPC teams to cover a broad range of the different domains of suffering (physical, psychological, social, and spiritual) [1, 3, 11, 30, 32]. Moreover, there is some evidence that SPC may have *economic benefits* without reducing quality of life [33, 34].

### Types of interventions

#### Definition of SPC and usual care

In this review, we will evaluate SPC services. We define SPC as described by Zimmermann et al.: “A specialized palliative care service was defined as a service of health care professionals from at least two different professions that provides or coordinates comprehensive care for patients with an [advanced illness]. Studies evaluating the impact of only 1 component of comprehensive palliative care on only 1 aspect of quality of life (eg, impact of pain medication on pain; impact of medication or psychotherapy on depression) will be excluded” [30]. Subsequently, we will use the criteria developed by Gomes et al. [30] to analyze the different SPC interventions of the included studies. SPC will be compared with usual care. We define usual care as care which is provided by personnel that are not designated as SPC professionals.

#### Site of care

We will evaluate SPC services in (i) hospitals (i.e., palliative care inpatient ward or hospital consulting services), (ii) hospices, or (iii) community settings.

#### Direct patient care

Services and interventions not directly delivering care to patients will not be included. This concerns education or training programs for health professionals as well as interventions limited to providing staff advice or offering service coordination. Moreover, interventions solely providing assessments (e.g., screening tool) or the implementation of new policies/algorithms and (e.g., national) strategies will not be included.

### Types of outcome measures

#### Primary outcome

- Patients’ QoL assessed with validated and non-validated questionnaires or single-items

#### Secondary outcomes

- Psychosocial variables: distress, depression, anxiety, spiritual well-being, social well-being, and satisfaction with care as reported by the patient and/or a proxy and assessed with validated and non-validated questionnaires or single-items
- Pain, fatigue, nausea, and dyspnea as reported by the patient and/or a proxy and assessed with validated and non-validated questionnaires or single-items
- Survival time
- Place of death (hospital, hospice, community setting, home, others)
- Cost of care (direct and indirect costs)
- Attrition number and reasons
- Adverse events

We will exclude studies that focus on outcomes other than those stated. Outcomes will be analyzed at the point in time of the primary outcome (except for the outcome place of death). Psychosocial variables and symptoms may differ whether they are reported directly by the patient or indirectly by a proxy. Therefore, they will be considered as two separate types of outcomes (e.g., constipation: patient-reported or proxy-reported).

### Search methods for identification of studies

#### Electronic searches

We will conduct searches using the databases MEDLINE (via Ovid), EMBASE (via DIMDI), Cochrane Central Register of Controlled Trials (CENTRAL), and PsycINFO (via EBSCO).

The search strategy that we will use to search MEDLINE can be found in Appendix 1. We will modify the search strategy where necessary to search the other databases listed. No date or language restrictions will be applied.

We applied the RCT sensitivity-maximizing search strategy provided by Cochrane [35] and parts of the BMJ search strategy for RCTs [36].

### Searching other resources

#### Textbooks and trial registers

We will hand-search the reference lists of the following three textbooks:

- Textbook of Palliative Medicine [37]
- Oxford Textbook of Palliative Medicine [38]
- Oxford Textbook of Palliative Nursing [39]

We will identify ongoing trials via (i) the metaRegister of controlled trials (http://www.controlled-trials.com/), (ii) clinicaltrials.gov (www.clinicaltrials.gov), and (iii) the WHO International Clinical Trails Registry Platform (ICTRP; http://apps.who.int/trialsearch/).

#### Reviews

We will identify relevant reviews and will hand-search the reference lists.

#### Correspondence

We will contact authors of the main studies and investigators who are known to be carrying out research about SPC and authors of relevant textbook sections and reviews for further studies and unpublished data. These will include at least (alphabetical order): Amy Abernethy, Susan Block, Andrew Billings, Eduardo Bruera, Stein Casa, David Currow, Barbara Gomes, Irene Higginson, John Lynch, Thomas J. Smith, Jennifer Temel, and Declan Walsh.

#### Conference proceedings and other

We will hand-search conference proceedings of the Congress of the European Association for Palliative Care (EAPC) 2013, 2014 and 2015.

### Data collection and analysis

#### Selection of studies

One review author (WS) will screen the titles and abstracts to remove duplicates. Two reviewers (JG, WS) will screen titles and abstracts for relevance and judge the eligibility of the studies. We will resolve disagreements by consensus and with a third review author (GB). Multiple reports of studies will be linked together. Uncertainties concerning multiple reporting will be resolved by contacting authors.

We will include a Preferred Reporting Items for Systematic Reviews and Meta-Analyses (PRISMA) flow chart [40] in the full review that will show the status of identified studies as recommended in the Cochrane Handbook [41].

#### Data extraction and management

We will enter data from each selected study into a data extraction form. Two reviewers (JG, WS) will independently extract data for each study. We will resolve disagreements by consensus or with a third review author (GB) when needed. We will contact authors of studies to provide unpublished data for the meta-analysis, if required.

#### Assessment of risk of bias in included studies

Two authors (JG, WS) will independently assess the risk of bias for each study. We will use the Cochrane Collaboration’s risk of bias tool and the corresponding criteria for judging risk of bias that are provided in the Cochrane Handbook [42]. The features of the Cochrane Collaboration’s risk of bias tool encompass the following:

- Selection bias: sequence generation and allocation concealment
- Performance bias: blinding of participants and personnel
- Detection bias: binding of outcome assessment
- Attrition bias: incomplete outcome data
- Reporting bias: selective outcome reporting
- Other bias: other sources of bias (e.g., not appropriate study design or contamination)

We will include these risk of bias assessments in the evaluation of evidence according to the Grading of Recommendations Assessment, Development, and Evaluation system (GRADE system) [42, 43]. The quality level concerning QoL (our primary outcome) or the primary outcome stated in a study will be down- or upgraded if applicable, according to the Cochrane Handbook [44]. Economic evidence will be assessed using the CHEERS-checklist [45].

#### Measures of treatment effect

The software Review Manager (Cochrane Collaboration) will be used to combine the data from all included studies. When sample size and proportions are missing, they will not be imputed or estimated for meta-analysis. Instead, we will contact the authors to acquire additional data. The random-effects model will be used for meta-analysis, since we anticipate that study designs and interventions will differ between studies. The Mantel-Haenszel (default configuration) will be used as the statistical method. If authors use the same assessment tools, we will evaluate continuous data using the mean difference (MD). If not, results from the meta-analysis will be shown using the standardized mean difference (SMD). Results for dichotomous data will be analyzed by calculating the risk ratio (RR). We will use the generic inverse-variance method to analyze survival data because it can integrate both log-rank and Cox model estimates. Moreover, the generic inverse-variance method enables a random-effects analysis whereas the “O – E and Variance” outcome type does not [41].

Hazard ratios and the corresponding standard errors will be calculated using methods described in Parmar et al. (1998) if not reported in publications [46].

To review the cost-saving potential of SPC, the total (cross-sectoral) cost of care for the SPC service group and the control group(s) will be retrieved from the eligible studies (if available) which provide an economic evaluation. To allow a consistent economic comparison, only those studies will be included in which palliative care is the intervention and in which cost measures are combined with QoL (i.e., economic information based on cost studies or cost of illness studies will not be extracted). Economic data should comprise hospital costs, other institutional care costs, community care costs, informal care costs, and equipment and medication prescribed. If only the cost for the additional SPC service is provided, this information will not be extracted. The reason is that this cannot be viewed as “additional costs” since SPC service provision may result in cost-saving effects in other health care sectors, e.g., due to fewer intensive or aggressive therapies (i.e., chemotherapy) or other procedures (e.g., diagnostic (staging) test) at the end of life [3].

#### Unit of analysis issues

We expect that the level of randomization may differ between studies. Individual randomization and cluster randomization is possible [11, 32]. We will take intra-cluster correlation (ICC) into account for studies reporting cluster randomization [32]. If ICC is not stated, the authors will be contacted. Repeated measurements for the same outcomes may also occur in trials. We will analyze outcomes at the point in time that was defined by the authors for the primary outcome (except for the outcome place of death). Other issues like individuals undergoing more than one intervention (e.g., cross-over trial) are extremely unlikely in the SPC study setting.

#### Dealing with missing data

We will attempt to contact the trial authors if relevant data (e.g., inadequate flowchart with reasons for dropouts) are missing. Numbers of screened and randomized patients as well as dropouts will be taken into account (e.g., sensitivity analysis). We will critically document whether the intention to treat (ITT) or per-protocol (PP) approach was used or if and which imputation methods (e.g., last observation carried forward) were applied. For trials using continuous outcomes in which standard deviations (SDs) were not reported and no information is available from the authors, we will calculate the SDs via the standard error of the mean (SEM).

#### Assessment of heterogeneity

The statistical heterogeneity will be judged by use of the chi-square test and *I*^2^ statistic [41]. *I*^2^ calculates the percentage of the variability in effect estimates due to heterogeneity rather than chance. Here, higher percentages suggest more observed heterogeneity with 0–40 % indicating not being important, 30–60 % representing moderate, 50–90 % substantial, and 75–100 % considerable heterogeneity [41].

Clinical heterogeneity will be assessed by analyzing results according to cancer type, age, early vs not early SPC, site of care, and setting (see also “Subgroup analysis and investigation of heterogeneity” section).

#### Assessment of reporting biases

Funnel plots of the primary outcome (QoL) will only be calculated to assess heterogeneity and publication bias if >10 studies are included in the meta-analysis [41]. We will use contour-enhanced funnel plots in order to facilitate differentiation between publication bias and other sources of asymmetry in the funnel plots [41]. Further, we will consider heterogeneity (patients, interventions, control group, study design) and contextual factors (e.g., historical or cultural factors) when interpreting funnel or forest plots.

In addition, reporting bias and publication bias will be assessed by checking the trial registers as stated in the chapter “Textbooks and trial registers.” Authors will be contacted if necessary (see also the “Correspondence” section).

#### Data synthesis

Summary of the evidence and quality appraisal for QoL or the primary outcome stated in a study will be presented according to the criteria developed by the GRADE working group [42, 43, 47]. We will generate a summary of findings table according to the GRADE system and include QoL (primary outcome) and the following secondary outcomes that were chosen in a consensus process and discussion in our research group: depression, satisfaction with care, pain, survival time, place of death, and number of patients with at least one adverse event.

In palliative care, health-related quality of life (HRQol) encompasses the physical, psychological, social, and spiritual domain. We will include all measures for QoL that include items from at least two of the four domains (physical, psychological, social, or spiritual) in our meta-analysis.

Data concerning the primary outcome (QoL) will be assessed using a random-effects model to account for the heterogeneity in populations and interventions [41]. We will use MDs or SMDs for continuous data and RRs for dichotomous data as effect estimates.

Statistical and clinical heterogeneity will be addressed as stated in the chapter “Assessment of heterogeneity” and “Assessment of reporting biases.”

### Subgroup analysis and investigation of heterogeneity

Considering previous studies [3, 18, 30], it is very likely that the participants, interventions, comparators, and outcomes will be highly variable (see the “Sensitivity analysis” section). If these are not judged as similar enough to ensure a clinically meaningful statement, meta-analysis will not be undertaken according to the Methodological Expectations of Cochrane Intervention Reviews (MECIR) standards [48]. This judgement will be a joint decision of the whole group. Results will be analyzed and presented in a descriptive way if no meta-analysis is performed.

We defined the following subgroups:

(i) Cancer vs non-cancer patients
(ii) Within non-cancer patients: patients with neurological disease (e.g., amyotrophic lateral sclerosis (ALS), multiple sclerosis (MS)) vs chronic-obstructive-pulmonary disease (COPD) vs heart failure
(iii) Elderly patients vs younger patients (elderly defined as >79 years of age according to the Medical Subject Heading (MeSH) “aged” and based on the clinical experience of the research group; younger patients are defined as 18–79 years)
(iv) Patients treated early vs not early with SPC (early defined as: Eastern Cooperative Oncology Group (ECOG) 0–2 or Karnofsky index 50–100 or 6–24 months estimated survival or initiation of SPC within 8 weeks after diagnosis of an advanced incurable illness as defined in the identified studies)
(v) Site of care: hospitals vs hospices vs community settings
(vi) Inpatients vs outpatients

We will analyze the subgroups using a random-effects model [49] and will evaluate the statistical heterogeneity using the chi-square test and *I*^2^ statistic (see the “Assessment of heterogeneity” section). For dichotomous data, we will use Mantel-Haenszel as statistical method because it has better statistical properties when there are few events [41].

If a meta-analysis is performed, the results should be interpreted with caution. A summary measure for the subgroups’ mean diamonds (total) will not be calculated if the study variability is too high (e.g., participants, interventions, etc.). If a meta-analysis is not possible, the results of the subgroups will be compared descriptively.

### Sensitivity analysis

As described earlier in the protocol, the studies eligible for this review will vary greatly in terms of the study population (underlying diagnosis, site of care, stage of the disease), intervention (teams with highly experienced and trained team members vs teams with rather unclear staff expertise), comparator (standard care at a large medical center vs standard care in a primary care setting), and study design (single-center vs multi-center studies). Concerning the outcome, no great variations are expected since most of the studies of previous reviews include QoL measures [3, 18, 30]. After primary data synthesis, we will identify the best and worst effect studies and critically judge them according to the mentioned factors (population, comparator, etc.). Assumptions and key issues of this group discussion will be recorded and reported. If applicable, sensitivity analysis will be undertaken after possibly misleading studies have been identified. We will publish a separate meta-analysis if the results change significantly after performing the sensitivity analysis for the mentioned factors (population, intervention, comparator, and study design). This sensitivity analysis is expected to identify areas for future research and possible subject-specific sources of bias that need to be taken into account by researchers when planning future clinical trials.

## Discussion

Within clinical and academic settings, the palliative care needs of patients have received increased attention and recognition, but still, these needs remain unmet for many patients [3, 8, 28]. Indeed, there is still a considerable degree of suffering among patients with advanced, incurable diseases and their families [16, 21, 23, 25, 26].

One way to improve this situation may be the integration of *SPC* teams into the care of patients with various advanced and incurable illnesses [3, 24, 28]. Landmark studies have reported an increased QoL, reduced patient and family distress, reduced aggressiveness of care and health care costs, and even the potential to prolong life [3, 27, 28].

To provide high-quality evidence, we have used Cochrane standards in this protocol. We have predefined how results will be evaluated, how heterogeneity will be considered, and how bias will be assessed.

Most importantly, we will pay close attention to capturing the specialist aspects and the holistic scope of palliative care interventions. Therefore, we have described patients, interventions, comparisons, and outcomes (PICO) precisely in this protocol. We will focus on patients with advanced malignant or non-malignant illness [31]. Caregivers will not be included as a second population in order to maintain a clear and well-defined study population and to closely follow the PICO framework [50].

We will use the SPC definition of Zimmermann et al. [30] because it reflects the multi-professional nature of the personnel (health care professionals from at least two different professions), and it assesses multi-dimensional outcomes in a pragmatic and efficient way: “Studies evaluating the impact of only 1 component of comprehensive palliative care on only 1 aspect of quality of life (eg, impact of pain medication on pain; impact of medication or psychotherapy on depression) were excluded” [30].

We chose QoL as our primary outcome. We acknowledge that pain intensity is an important outcome and has a lower risk of heterogeneity than QoL. However, SPC interventions should offer a multi-dimensional approach, according to the definition of the WHO. For example, interventions primarily aimed at reducing pain (physical domain) would be considered a part of palliative care but not palliative care itself. We prefer QoL as our primary outcome because it captures bio-psycho-social-spiritual aspects and represents the core of palliative care, despite the fact that no universal understanding of the concept or even consensus definition of QoL exists [51]. In palliative care, QoL is usually related to symptom control, physical function, social functioning, psychological well-being, meaning, and fulfilment (existential and spiritual aspects) and is referred to as HRQol. Therefore, we decided to include items from at least two of the four QoL domains (physical, psychological, social, or spiritual) in our meta-analysis (if applicable).

Evidence from RCTs on this subject will be identified, and the resulting body of evidence will be analyzed as described in the methods chapter. Our results will provide an overview of potential effects of SPC and may provide a useful basis for decision-making among health care providers and policy makers.

## Appendix

**Table 1:** Ovid MEDLINE search strategy

#	Search
1.	*Palliative Care/
2.	palliative care.ab,ti.
3.	support* care.ab,ti.
4.	early palliative care.af.
5.	special* palliative care.af.
6.	terminal care.ab,ti.
7.	hospice.ab,ti.
8.	coordinat* care.ab,ti.
9.	comprehensiv* care.ab,ti.
10.	1 or 2 or 3 or 4 or 5 or 6 or 7 or 8 or 9
11.	randomized controlled trial.pt.
12.	controlled clinical trial.pt.
13.	randomized.ab.
14.	placebo.ab.
15.	clinical trials as topic.sh.
16.	randomly.ab.
17.	trial.ti.
18.	11 or 12 or 13 or 14 or 15 or 16 or 17
19.	((comment or editorial or meta-analysis or practice-guideline or review or letter or journal correspondence) not “randomized controlled trial”).pt.
20.	(random sampl$ or random digit$ or random effect$ or random survey or random regression).ti,ab. not “randomized controlled trial”.pt.
21.	exp animals/ not humans.sh.
22.	child*.mp. or Child/
23.	10 and 18
24.	19 not (19 or 20 or 21 or 22)

## Additional file

Additional file 1:
**Preferred Reporting Items for Systematic review and Meta-Analysis Protocols (PRISMA-P) 2015 checklist: recommended items to address in a systematic review protocol.**

## Abbreviations

EAPC: European Association for Palliative Care
ECOG: Eastern Cooperative Oncology Group
GB: Gerhild Becker
GRADE: Grading of Recommendations Assessment, Development and Evaluation
HRQol: health-related quality of life
ICC: intra-cluster correlation
ITT: intention to treat
JG: Jan Gaertner
MD: mean difference
PICO: patients, intervention, comparison and outcomes
PP: per-protocol
QoL: quality of life
RCT: randomized controlled trial
SD: standard deviation
SEM: standard error of the mean
SMD: standardized mean difference
SPC: specialist palliative care
WHO: World Health Organization
WS: Waldemar Siemens
